# miR-362-5p promotes cell proliferation and cell cycle progression by targeting GAS7 in acute myeloid leukemia

**DOI:** 10.1007/s13577-019-00319-4

**Published:** 2020-01-10

**Authors:** Fuqun Wu, Changxin Yin, Junhua Qi, Deyu Duan, Xi Jiang, Jianhua Yu, Zhaofan Luo

**Affiliations:** 1 Department of Clinical Laboratory, The Seventh Affiliated Hospital of Sun-Yat-Sen University, No. 628, Zhenyuan Road, Guangming District, Shenzhen, 518017 Guangdong China; 2 Department of Hematology, Kanghua Hospital, Dongguan, 523080 Guangdong China; 3 Department of Hematology, Nanfang Hospital, Southern Medical University, Guangzhou, 510515 Guangdong China

**Keywords:** Acute myeloid leukemia, miR-362-5p, GAS7, Cell cycle

## Abstract

Recently, miR-362-5p has attracted special interest as a novel prognostic predictor in acute myeloid leukemia (AML). However, its biological function and underlying molecular mechanism in AML remain to be further defined. Herein, we found that a significant increase in miR-362-5p expression was observed in AML patients and cell lines using quantitative real-time PCR. The expression of miR-362-5p was altered in THP-1 and HL-60 cells by transfecting with miR-362-5p mimic or inhibitor. A series of experiments showed that inhibition of miR-362-5p expression significantly suppressed cell proliferation, induced G0/G1 phase arrest and attenuated tumor growth in vivo. On the contrary, ectopic expression of miR-362-5p resulted in enhanced cell proliferation, cell cycle progression and tumor growth. Moreover, growth arrest-specific 7 (GAS7) was confirmed as a direct target gene of miR-362-5p and was negatively modulated by miR-362-5p. GAS7 overexpression imitated the tumor suppressive effect of silenced miR-362-5p on THP-1 cells. Furthermore, miR-362-5p knockdown or GAS7 overexpression obviously down-regulated the expression levels of PCNA, CDK4 and cyclin D1, but up-regulated p21 expression. Collectively, our findings demonstrate that miR-362-5p exerts oncogenic effects in AML by directly targeting GAS7, which might provide a promising therapeutic target for AML.

**Electronic supplementary material:**

The online version of this article (10.1007/s13577-019-00319-4) contains supplementary material, which is available to authorized users.

## Introduction

Human leukemias are a genetically and clinically heterogenous group of diseases characterized by malignant proliferation of hematopoietic progenitors and impaired function of normal hematopoietic [1]. With an overall incidence of 3.4 cases per 100,000 inhabitants in the United States, acute myeloid leukemia (AML) is one of the most common types of leukemia in adults and can rise “de novo” or as a secondary event [2]. Though chemotherapeutic agents, molecular targeted drugs, and hematopoietic stem cell transplantation have shown therapeutic efficacy in AML, many patients still relapse [2–4]. Nowadays, epigenetic alterations are becoming increasingly valued for their contribution to hematopoietic transformation likely by providing growth advantages and altering expression of cancer specification genes [3, 5]. Therefore, a deep understanding of epigenetic mechanisms and dysregulations in AML will become a priority in biomedical research.

Notably, evolutionarily conserved short non-coding single-stranded RNAs of 19–23 nucleotides, collectively named microRNAs (miRNAs), have been characterized as negative regulators of mRNA stability [6, 7]. It was shown that miRNAs could bind to their target mRNA through RNA–RNA base pairing, frequently resulting in reduction of the levels of their target transcripts and the amount of protein encoded by these transcripts [8]. miRNAs can act as oncogenes or anti-oncogenes, facilitating cancer initiation and progression in solid and hematological malignancies [9]. Functionally, the implication of several miRNAs in fundamental biological processes has been identified in AML, such as apoptosis (miR-29b [10] and miR-149-5p [11]), differentiation (miR-155 [12]), cell cycle and proliferation (miR-125a [13] and miR-192 [14]). Recently, the distorted and unique expression pattern of miR-362-5p in several types of malignant tumor coupled with its abnormal expression in keeping the malignant phenotype of cancer cells make miR-362-5p a novel molecular target for regulation and detection [15–17]. miR-362-5p was described as a tumor inducer in breast cancer [16], chronic myelocytic [17], and hepatocellular carcinoma [18] and its amplification, by releasing the promotion upon tumor-inhibiting genes such as CYLD and GADD45α, promotes tumorigenesis and metastasis. Conversely, miR-362-5p is down-regulated in neuroblastoma and by targeting phosphatidylinositol 3-kinase-C2β, inhibits cell proliferation and migration [15]. Ma et al. showed that miR-362-5p is a novel predictor for prognosis of patients with cytogenetically normal AML [19]. While, the precise function of miR-362-5p is still unknown in AML and needs further investigation.

GAS7 is a growth arrest-specific gene first isolated from the serum-starved cells, NIH3T3 [20]. It is a member of the pombe Cdc 15 homology family (PCH) that belongs to the subfamily of proline, serine, threonine-rich phosphatase interacting proteins (PSTPIP) [21]. All three distinct isoforms of the human GAS7 (GAS7a, GAS7b, and GAS7c) contain an N-terminal Fes/CIP4 homology (FCH) domain, and these isoforms have slightly different orientations [21]. Brain tissues have been reported to express GAS7 and are essential for morphological differentiation and neuritogenesis in both human and rodent cells [22]. It is notable that the anti-cancer action of GAS7 in lung squamous cell carcinoma (LSCC) was proposed by Tseng et al. [23]. To date, GAS7 is negatively associated with non-small cell lung cancer (NSCLC) phenotype and it is under-expressed in a large cohort of patients undergoing NSCLC [24]. Several previous studies have reported the relationship between a representative MLL fusion protein, MLL-GAS7, and leukemias. MLL-GAS7 can impair the differentiation of the hematopoietic progenitors and induce mixed lineage leukemias in mice [25, 26].

In this study, we shed light on the detailed miR-362-5p expression patterns of blood samples derived from AML patients and normal controls, as well as AML cell lines and normal bone marrow cell line (HS-5). The functional and mechanistic aspects of miR-362-5p on the progression of AML were evaluated in vitro and in vivo. A series of experiments were carried out to determine whether GAS7 was a direct target of miR-362-5p in AML THP-1 cells. These studies help provide an advanced knowledge of miR-362-5p/GAS7 network in the malignant progression of AML.

## Materials and methods

### Clinical tissues collection

A total of 24 fresh blood samples were collected from patients diagnosed as AML (age range, 28–72 years; 10 female, 14 male) by bone marrow aspiration and biopsy at the Seventh Affiliated Hospital of Sun Yat-Sen University (Shenzhen, China) between September 2016 and March 2018. Meanwhile, the normal samples (*n* = 24, age range, 37–58 years; 12 female, 12 male) were also collected from healthy volunteers as the control group. Prior to enrollment, each participant signed informed consent. The study was performed in accordance with the Declaration of Helsinki and obtained the approval from the Ethics Committee of Seventh Affiliated Hospital of Sun Yat-Sen University.

### Cell culture

AML cell lines (TF-1, HL-60 and THP-1) and normal HS-5 cells were obtained from American Type Culture Collection (ATCC, Manassas, VA, USA). All cell lines were cultured in RPMI-1640 with 10% fetal bovine serum (FBS) (all from Gibco, CA, USA) and maintained in a humidified incubator containing 5% CO_2_ at 37 °C.

### Quantitative real-time PCR

Total RNA was extracted using the miRNeasy extraction kit (Qiagen, Valencia, CA, USA). Quantification of miR-362-5p was performed using a Hairpin-it™ miRNA qPCR Quantitation Kit (GenePharma, Shanghai, China) with U6 small nuclear RNA gene (U6 snRNA) as an internal control. Relative mRNA levels of GAS7 were determined using Power SYBR Green PCR Master Mix (Applied Biosystems) with GAPDH as an internal control under the following conditions: 2 min at 50 °C, 10 min at 95 °C, 15 s at 95 °C, and 1 min at 60 °C for 40 cycles. All PCR reactions were performed on 7500 Fast Real-Time PCR Systems (Applied Biosystems, CA, USA). The sequences of the primer used are listed in Table 1. Relative quantification of miR-362-5p or GAS7 was calculated using the 2^−ΔΔCT^ method [27].

**Table 1:** Primers used for quantitative real-time PCR analysis

Gene	Primer sequence 5′-3′
miR-362-5p	F: AATCCTTGGAACCTAGGTGTGAGTAA R: ATCCTTGGAACCTAGGTGTGAGT
U6	F: CTCGCTTCGGCAGCACA R: AACGCTTCACGAATTTGCGT
GAS7	F: CGAGCTACGTGCAGTTGCT R: CATGTGGGCAGTCTCTGGAG
β-actin	F: GGCGGCACCACCATGTACCCT R: AGGGGCCGGACTCGTCATACT

*F* forward, *R* reverse

### Cell transfection

miR-362-5p inhibitor, miR-362-5p mimic and negative control (miR-NC) oligonucleotides were provided by RiboBio Co., Ltd. (Guangzhou, China). THP-1 or HL-60 cells were seeded into six-well plates and transfected with miR-362-5p inhibitor, miR-362-5p mimic or miR-NC, respectively, at a final concentration of 50 nM using Lipofectamine 2000 (Invitrogen, Carlsbad, CA, USA). Full-length cDNA for human AGS7 was obtained, amplified and cloned into pcDNA3.1 expression vector GenePharma (Shanghai, China). AGS7 overexpression was accomplished by transfection of AGS7 plasmid or empty vector with Lipofectamine 2000. The following in vitro experiments were conducted 48 h after transfection.

### CCK-8 assay

THP-1 or HL-60 cells at a density of 2 × 10^4^ cell per well were seeded in 96-well plates in triplicates. Cell proliferation was evaluated using the Cell Counting Kit-8 (CCK-8) Assay kit (Dojindo Molecular Technologies Inc, Kumamoto, Japan) according to the manufacturer’s protocol. In brief, cells were incubated in 10% CCK-8 reagent at 37 °C for 2 h at indicated time points. The absorbance at a wavelength of 450 nm was determined using a microplate reader (Bio-Tek, VY, USA).

### Cell cycle analysis

Cell cycle distribution was analyzed by propidium iodide (PI) staining, followed by flow cytometry analysis. Briefly, THP-1 or HL-60 cells were harvested, washed with PBS twice and re-suspended in RPMI-1640 at a concentration of 3 × 10^5^ cells per well. Then, the cells were fixed with 70% ethanol for 1 h at 4 °C and incubated with 50 µL of RNase 1 and 25 µL of propidium iodide solution (both from BioLegend, San Diego, CA, USA). DNA histograms for cell cycle were determined using a flow cytometer (FACSCanto™ II, BD Biosciences, Franklin Lakes, NJ, USA).

### Luciferase reporter assay

The wild-type GAS7 3′UTR containing the predicted binding site for miR-362-5p from TargetScan online database (targetscan.org/vert_71) was cloned into the luciferase vector psi-CHECK2 (Promega, Madison, USA), referred to as WT GAS7. The mutant GAS7 3′UTR was constructed using Q5^®^ site-directed mutagenesis kit (E0554S, Biolabs) and also inserted into psi-CHECK2 to form MUT GAS7. For luciferase reporter assay, THP-1 cells at a density of 1 × 10^5^ cells/well were plated in 96-well plates. Next, we used Lipofectamine 2000 to transfect THP-1 cells with WT GAS7 or MUT GAS7 together with miR-362-5p mimic, miR-362-5p inhibitor or miR-NC for 48 h. The firefly and Renilla luciferase activities were measured using the Dual-Luciferase Reporter Assay (Promega) and relative luciferase activities were calculated.

### Western blot analysis

Total cellular protein was extracted from THP-1 cells using RIPA lysis buffer (Beyotime Biotechnology, Shanghai, China). After protein quantification with a BCA protein assay kit (Beyotime Biotechnology), equal amounts of protein were electrophoresed on 10% SDS-PAGE and transferred to polyvinylidene difluoride (PVDF) membranes (Millipore, MA, USA). Next, the membranes were blocked with 5% non-fat milk in Tris-buffered saline containing 0.1% Tween-20 (TBST) and incubated overnight at 4  °C with primary antibodies against GAS7, PCNA, CDK4, Cyclin D1, p21 and GAPDH. Following washing with TBST three times, the membranes were incubated with the corresponding horseradish peroxidase-conjugated secondary antibodies for 2 h at room temperature. All protein signals were detected using enhanced chemiluminescence kit (ECL; Bio-Rad Laboratories, Inc., Hercules, CA, USA).

### Tumor xenograft experiments

BALB/c nude mice (4–6 weeks) were purchased from the Animal Resources Centre (Guangdong, China) and maintained in specific pathogen-free cages with a 12-h light/dark cycle. Three groups of THP-1 cells were prepared, including stably expressing miR-362-5p mimic, miR-362-5p inhibitor or miR-NC, respectively. Then, approximately 1 × 10^6^ THP-1 cells in 200 μL PBS were injected subcutaneously into the right posterior flank of each nude mouse. Every 5 days, tumor growth, including length (*L*) and width (*W*) was measured and tumor volumes were calculated by the formula: volume (cm^3^) = (*L* × *W*^2^)/2. Based on the average tumor volume, growth curves in each group were drawn. After 40 days, the mice were sacrificed and tumors were weighed and dissected. The dissected tumors were fixed in 10% neutralized formalin overnight for GAS7 and CDK4 expression examination. All procedures were conducted in accordance with the Declaration of Helsinki and approved by the Animal Care and Use committee of the Seventh Affiliated Hospital of Sun Yat-Sen University.

### Immunohistochemistry (IHC)

The formalin-fixed tumors were dehydrated, embedded in paraffin, cut into 4 μm sections and subjected to IHC staining with specific antibodies against GAS7 or CDK4 using standard techniques. After incubation with horseradish peroxidase labeled secondary antibody, the protein expression was visualized and evaluated based on the percentage of positive cells and the intensity of staining by two independent pathologists who were blinded to this study.

### Statistical analysis

Statistical analyses were performed using SPSS (version 23.0, SPSS Inc.). Data were expressed as mean value ± standard deviation (SD) of three independent determinations. Student’s *t* test or one-way ANOVA, followed by the Bonferroni multiple comparison test was used for comparison between two groups or multiple groups, respectively. The correlation between miR-362-5p and GAS7 expression in AML samples was determined using Pearson’s correlation coefficient. A *p* value of less than 0.05 was considered statistically significant.

## Results

### miR-362-5p expression was increased in AML clinical samples and cell lines

**Fig. 1:**
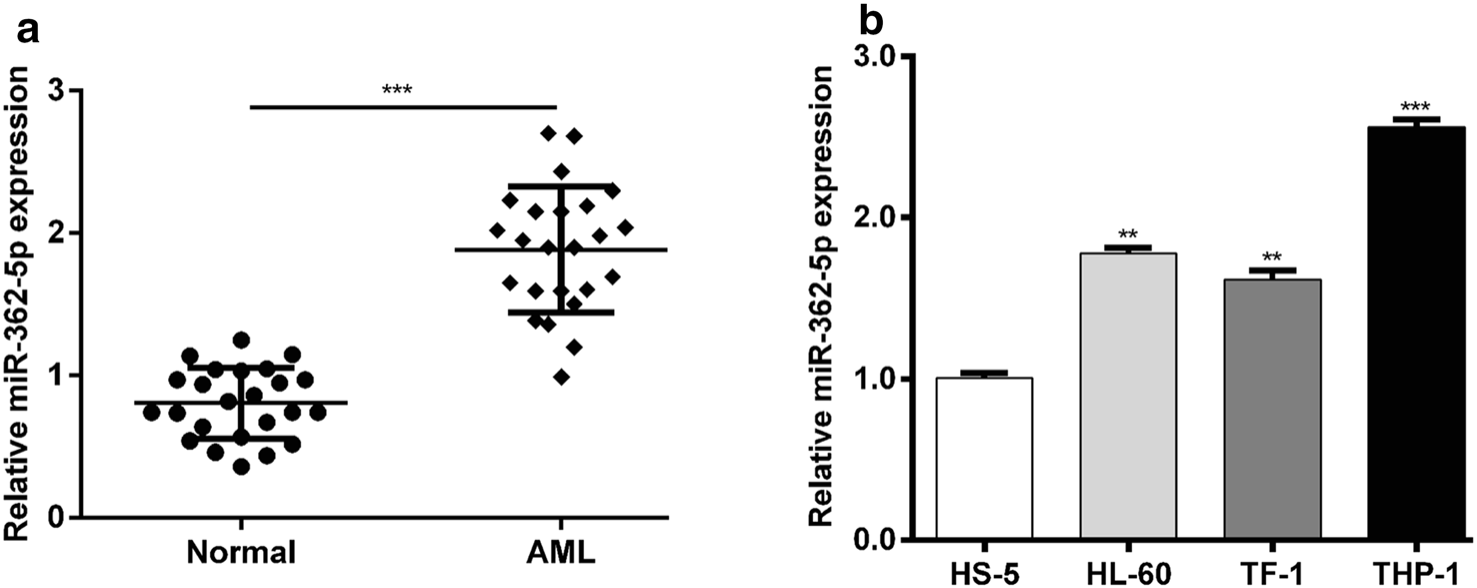
miR-362-5p expression was up-regulated in AML. **a** Quantitative real-time PCR analysis was performed to detect miR-362-5p expression in the blood samples derived from 24 patients with AML and normal controls. ***p < 0.001 versus normal controls; **b** The expression status of miR-362-5p in three human AML cell lines (HL-60, TF-1, and THP-1) and a normal bone marrow cell line (HS-5) was determined with quantitative real-time PCR analysis. Each value represents mean ± SD in three independent experiments. **p < 0.01, ***p < 0.001 versus HS-5

To investigate the biological role of miR-362-5p in AML, the expression level of miR-362-5p was first determined in blood samples derived from 24 patients with AML. Quantitative real-time PCR demonstrated a dramatic increase of miR-362-5p expression in AML samples compared with normal samples (Fig. 1a, *p* < 0.001). In addition, we measured its expression in three AML cell lines and found that miR-362-5p was significantly up-regulated when compared to the normal cell line HS-5 (Fig. 1b, *p* < 0.01, *p* < 0.001). The data indicate that up-regulated miR-362-5p may play an oncogenic role in the progression of AML.

### miR-362-5p promoted AML cell proliferation and cell cycle progression in vitro

**Fig. 2:**
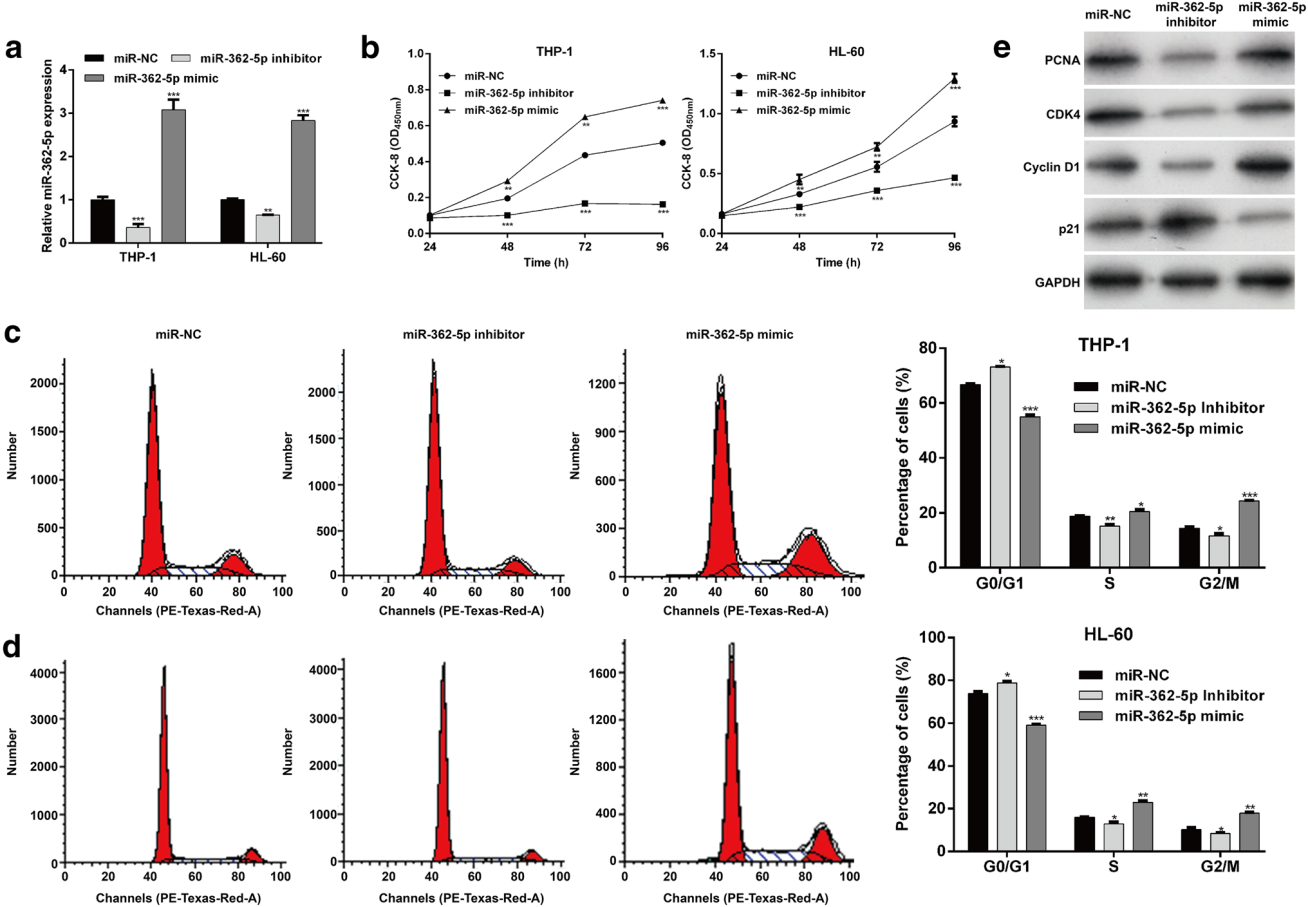
miR-362-5p promoted cell proliferation and cell cycle progression in THP-1 cells. **a** Quantitative real-time PCR analysis was performed to measure miR-362-5p expression in THP-1 and HL-60 cells after transfection with miR-362-5p inhibitor, mimic or miR-NC. **b** CCK-8 assay was used to evaluate the proliferative ability in THP-1 and HL-60 cells after transfection with miR-362-5p inhibitor, mimic or miR-NC. **c**, **d** Cell cycle status of THP-1 and HL-60 cells treated with miR-362-5p inhibitor, mimic or miR-NC was determined using flow cytometry analysis. Each value represents mean ± SD in three independent experiments. *p < 0.05, **p < 0.01, ***p < 0.001 versus miR-NC; **e** THP-1 cells after transfection with miR-362-5p inhibitor, mimic or miR-NC were subjected to western blot analysis for PCNA, CDK4, cyclin D1 and p21. GAPDH was used as internal control

THP-1 and HL-60 cells exhibited relatively higher miR-362-5p expression among the three AML cell lines, thus were selected as in vitro model to investigate the effects of miR-362-5p on AML progression. At first, quantitative real-time PCR confirmed miR-362-5p expression significantly decreased after miR-362-5p inhibitor transfection, but increased after miR-362-5p mimic transfection in THP-1 and HL-60 cells (Fig. 2a, *p* < 0.001). Using CCK-8 assay, we found miR-362-5p knockdown was significantly suppressed, but overexpression obviously promoted cell proliferation at the indicated time points (48, 72 or 96 h, respectively) in THP-1 and HL-60 cells (Fig. 2b, *p* < 0.01, *p* < 0.001). Furthermore, we investigated whether cell cycle distribution was affected in transfected THP-1 and HL-60 cells. As illustrated in Fig. 2c, the percentage of cells in G0/G1 phase was significantly elevated in miR-362-5p inhibitor group (73.13% ± 0.24%, *p* < 0.05), but reduced in miR-362-5p mimic group (54.99% ± 0.80%, *p* < 0.001), in comparison with miR-NC group (66.76% ± 0.31%). Accordingly, the percentage of cells in S and G2/M phase was remarkably decreased in miR-362-5p inhibitor group (*p* < 0.05, *p* < 0.01), but increased in miR-362-5p mimic group (*p* < 0.05, *p* < 0.001) compared with miR-NC group. Flow cytometry analysis of DNA histograms confirmed the observations of G0/G1 cell cycle arrest after miR-362-5p knockdown. Similarly, we found down-regulation of miR-362-5p-induced cell cycle G0/G1 phase arrest, which was reversed by miR-362-5p overexpression in HL-60 cells (Fig. 2d). We further evaluated the protein levels associated with miR-362-5p knockdown-mediated growth inhibition and G0/G1 arrest in THP-1 cells. As demonstrated in Fig. 2e, PCNA, CDK4 and Cyclin D1 were notably reduced, associated with up-regulation of CDK inhibitor p21 in THP-1 cells expressing miR-362-5p inhibitor, which was significantly reversed by miR-362-5p mimic transfection. Collectively, these results suggest that miR-362-5p may play a positive role in AML cell proliferation and cell cycle progression.

### GAS7 was a direct target gene of miR-362-5p

**Fig. 3:**
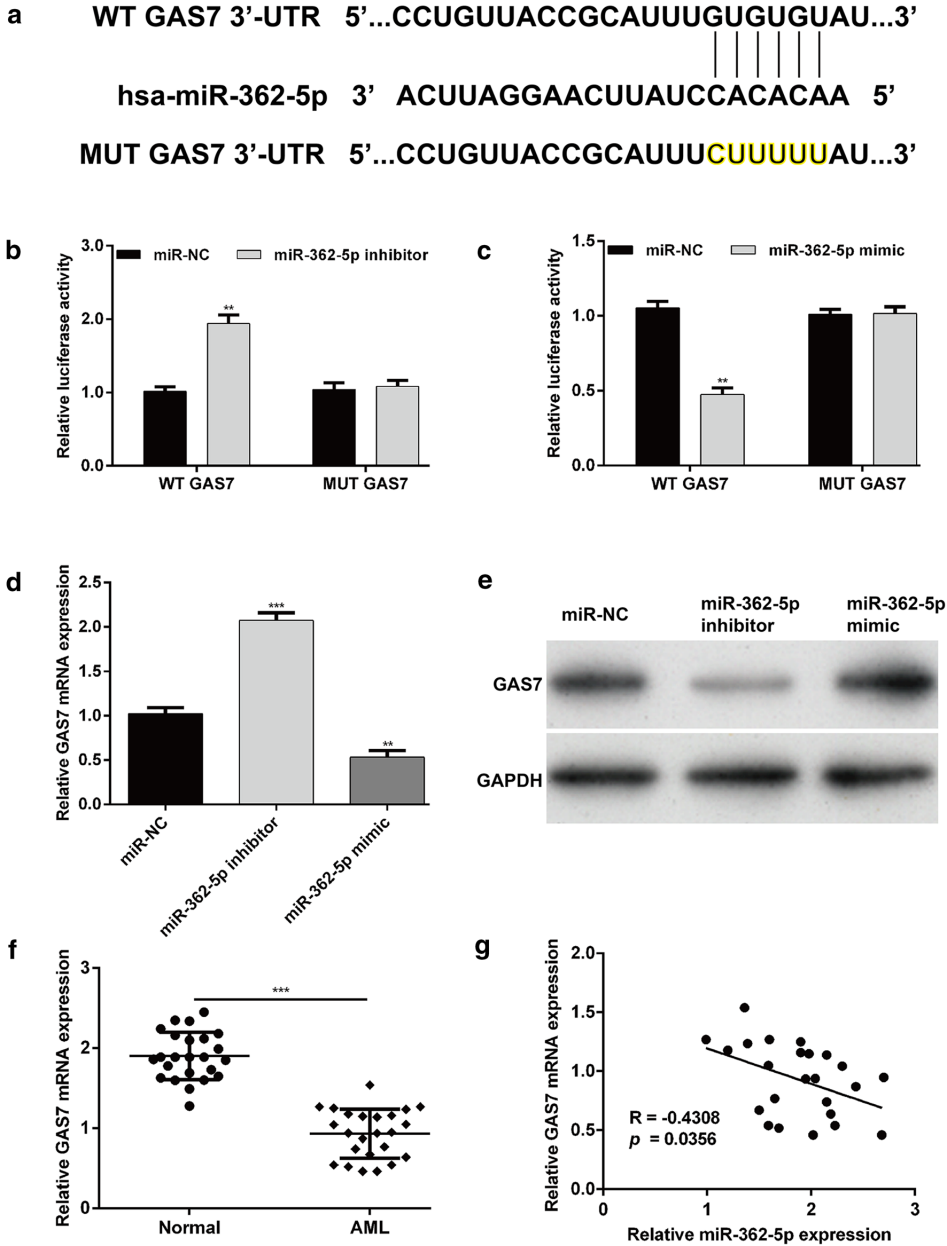
miR-362-5p directly targets GAS7 in THP-1 cells. **a** The putative wild-type (WT) and mutated (MUT) binding sites for miR-362-5p in the 3′-UTR of GAS7 are shown. **b**, **c** miR-362-5p inhibitor, mimic or miR-NC and a luciferase plasmid carrying the WT or MUT GAS7 were transfected into THP-1 cells. After 48 h of transfection, the transfected cells were harvested and subjected to quantification of luciferase activity using a Dual-Luciferase Reporter Assay System. **d**, **e** Quantitative real-time PCR and western blot analysis were performed to detect the expression levels of GAS7 mRNA and protein, respectively, in miR-362-5p knockdown or overexpression THP-1 cells. **f** The relative expression levels of GAS7 mRNA in the blood samples derived from 24 patients with AMI and normal controls. **g** The Pearson’s correlation analysis for the association between miR-362-5p levels and GAS7 mRNA levels in AML. Each value represents mean ± SD in three independent experiments. *p < 0.05, **p < 0.01, ***p < 0.001 versus miR-NC

To explore the mechanisms underlying the role of miR-362-5p in AML cell proliferation, the potential target genes of miR-362-5p were predicted using the TargetScan online database. Among these predicted target genes, GAS7 was of particular interest because of its involvement in AML progression [25, 26, 28]. As described in Fig. 3a, the 3′UTR of GAS7 contains a highly conserved binding site for miR-362-5p. Luciferase reporter assay was performed to validate whether GAS7 is a direct target of miR-362-5p. The results showed that miR-362-5p knockdown increased (Fig. 3b, *p* < 0.01) while overexpression decreased (Fig. 3c, *p* < 0.01) the luciferase activity in THP-1 cells transfected with the WT GAS7 but not the activity of the MUT GAS7. We next examined whether miR-362-5p affected the endogenous GAS7 expression in THP-1 cells. Quantitative real-time PCR (Fig. 3d, *p* < 0.01, *p* < 0.001) and western blot analysis (Fig. 3e) revealed that knockdown of miR-362-5p expression significantly increased the expression of GAS7, whereas enforced miR-362-5p decreased the GAS7 expression in THP-1 cells. In addition, the expression of GAS7 mRNA was significantly up-regulated in AML samples compared with normal samples (Fig. 3f). Pearson’s correlation analysis further demonstrated that the expression of miR-362-5p was inversely correlated with the GAS7 mRNA levels in AML samples (Fig. 3g). These observations indicate that miR-362-5p negatively regulated GAS7 expression in AML cells through binding its 3′UTR sequence.

### Overexpression of GAS7 expression imitated the effects of miR-362-5p knockdown in AML cells

**Fig. 4:**
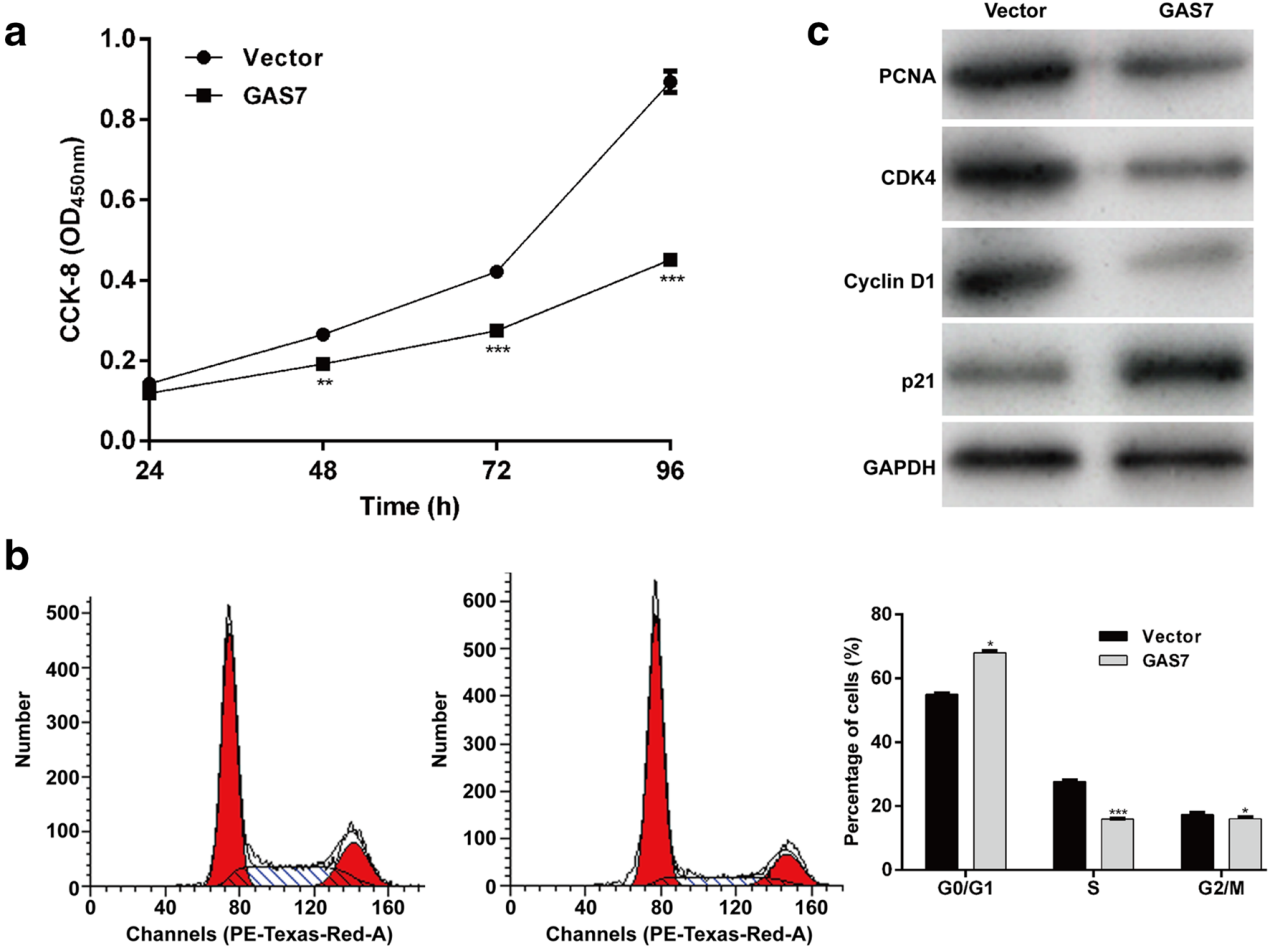
Overexpression of GAS7 expression imitated the effects of miR-362-5p knockdown in THP-1 cells. **a** The effect of GAS7 overexpression on THP-1 cell proliferation was determined by CCK-8 assay. **b** Flow cytometry analysis was used to assess the cell cycle status of THP-1 cells after transfection with GAS7 expression plasmid pcDNA3.1-GAS7 or empty vector. Each value represents mean ± SD in three independent experiments. *p < 0.05, **p < 0.01, ***p < 0.001 versus vector; **c** THP-1 cells after transfection with pcDNA3.1-GAS7 or empty vector were subjected to western blot analysis for PCNA, CDK4, cyclin D1 and p21. GAPDH was used as internal control

Considering GAS7 as a direct target gene of miR-362-5p, we next explored the functional role of GAS7 by transfecting GAS7 expression plasmid pcDNA3.1-GAS7 in THP-1 cells. CCK-8 assay indicated that GAS7 overexpression significantly reduced the proliferation of THP-1 cells (Fig. 4a, *p* < 0.01, *p* < 0.001). In addition, transfection of pcDNA3.1-GAS7 in THP-1 cells induced a significant increase in the percentage of cells at G0/G1 phase (*p* < 0.05) and decrease in the percentage of cells at S (*p* < 0.001) and G2/M phase (*p* < 0.05) compared with those in empty vector transfection (Fig. 4b). Moreover, we found overexpression of GAS7 notably suppressed the expression of PCNA, CDK4 and Cyclin D1, but enhanced p21 expression in THP-1 cells (Fig. 4c). Taken together, these data demonstrated that GAS7 overexpression could mimic the inhibition of miR-362-5p knockdown.

### miR-362-5p promoted tumor growth in AML in vivo

**Fig. 5:**
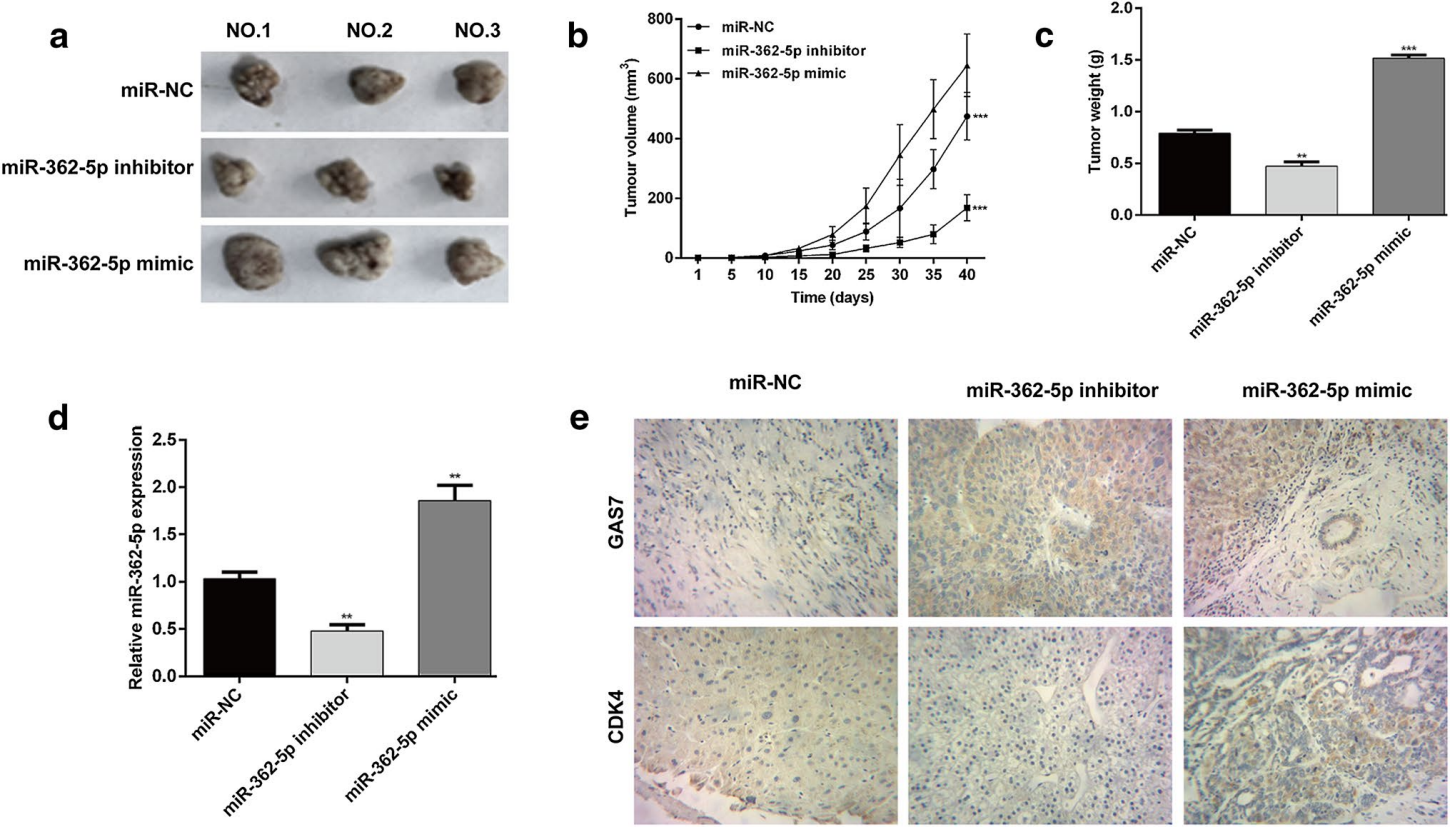
miR-362-5p promoted the growth of AML tumors in vivo. **a** Representative images of the xenograft tumors obtained from miR-362-5p inhibitor, miR-362-5p mimic or miR-NC-transfected THP-1 cells. **b** The tumor volume was detected every 5 days for 40 days. The tumor volumes were calculated in three groups. **c** The xenograft tumors formed were excised after 40 days. The weights were compared in three groups. **d** The expression level of miR-362-5p in the tumor xenografts was detected with quantitative real-time PCR. Each value represents mean ± SD in three independent experiments. **p < 0.01, ***p < 0.001 versus miR-NC; **e** expression of GAS7 and CDK4 proteins was detected in each group by IHC (400 ×) in the tumor xenografts

To validate the oncogenic role of miR-362-5p in AML progression, we inoculated miR-362-5p inhibitor, miR-362-5p mimic or miR-NC transfected THP-1 cells into nude mice. As shown in Fig. 5a, obvious differences were observed in tumor formation size in miR-NC, miR-362-5p inhibitor and miR-362-5p mimic groups. Every 5 days, the volume of xenografts formed tumor was detected and the results showed that inoculated tumors grew more rapidly in miR-362-5p mimic group, slowly in miR-362-5p inhibitor group, in comparison with miR-NC group (Fig. 5b, *p* < 0.001). Similarly, the tumor weight of miR-362-5p mimic group was significantly increased (*p* < 0.001), while that of miR-362-5p inhibitor group (*p* < 0.01) was decreased, compared with that of miR-NC group (Fig. 5c). To confirm miR-362-5p down-regulation was responsible for the in vivo tumor growth suppression, quantitative real-time PCR analysis was performed to measure the expression level of miR-362-5p in tumor xenografts. As shown in Fig. 5d, miR-362-5p was expressed at higher and lower levels in the tumor xenografts obtained from miR-362-5p mimic (*p* < 0.01) and inhibitor group (*p* < 0.01), respectively. Furthermore, IHC was performed in all groups to assess the protein expression of GAS7 and CDK4 (Fig. 5e). Consistent with the in vitro results, THP-1 tumor cells exhibited high expression of GAS7 protein and low expression of CDK4 after treatment with miR-362-5p inhibitor, which was notably reversed after treatment with miR-362-5p mimic. Overall, these observations suggest that miR-362-5p promoted the growth of AML cells in vivo via direct targeting of GAS7.

## Discussion

In this study, we aimed to investigate the role of miR-362-5p in AML cell proliferation and cell cycle progression. Here, miR-362-5p was found to be up-regulated in leukemia cell lines and blood samples of AML patients compared to that of the normal controls. This particular miRNA has exhibited its overexpression in breast cancer [16], chronic myelocytic [17], and hepatocellular carcinoma [18], whereas the same miRNA was reported to be under-expressed in neuroblastoma [15].

Accumulating evidence shows that dysregulation of miRNAs is closely associated with tumorigenesis and metastasis [7]. Expectably, we observed that inhibition of miR-362-5p in THP-1 cells could affect several characteristics related to cells growth, including decreased proliferative capacity and increased G0/G1 cell cycle arrest, whereas miR-362-5p overexpression has the opposite effect. Using an established tumor xenograft model, we showed that miR-362-5p mimic treatment increased tumor formation, while miR-362-5p inhibitor produced a marked reduction in the tumor formation. The data generated from hepatocellular carcinoma cell lines functional analysis indicated that the tumor growth and metastasis were induced by miR-362-5p [18]. A significant and positive correlation between overexpression of miR-362-5p and malignancy was observed in breast cancer cells and chronic myelocytic cells [16, 17]. Notably, in a previous study based on the prognostic value of miR-362-5p in AML, the survival of high expressers was shorter than the patients with low or absent miR-362-5p expression [19]. Our observations suggested that uncontrolled cell proliferation and impaired cell cycle progression are the main contributing factors of short overall survival in high miR-362-5p expression.

It has been shown that abnormal expression of a single miRNAs may have a profound impact on the expression profiles of multiple mRNAs which accelerates the cells towards transformation [29, 30]. To explore the molecular mechanisms by which miR-362-5p impacts THP-1 cells proliferation and cell cycle distribution, the potential target of miR-362-5p was predicted and verified in vitro. Among the predicted target genes of miR-362-5p, GAS7 was of particular interest because of its involvement in AML progression [25, 26, 28], which was thus selected as a potential target gene of miR-362-5p for further analysis. Our data further demonstrated that the expression of miR-362-5p was inversely correlated with the GAS7 mRNA levels in AML samples. Enhancing expression of GAS7 closely imitated the effects of miR-362-5p knockdown in THP-1 cells. Western blot analysis of PCNA and cell cycle regulators (CDK4, Cyclin D1 and the universal inhibitor of cyclin kinases, p21) in miR-362-5p-inhibitor or GAS7 transfected cells showed a decrease in PCNA, CDK4, and cyclin D1 levels and an increase in p21 levels. PCNA, which is a nuclear protein, is implicated in DNA synthesis and repair [31]. The expression of PCNA is often used to indicate proliferative activity [32]. It is known that the complex formed by CDK4 and cyclin D1 could promote cell cycle progression through G1/S transition, and the down-regulation of the complex corresponds well with G0/G1 phase arrest [33]. By regulating the four genes, GAS7 was found to inhibit THP-1 cells proliferation and induce G0/G1 phase arrest, and is directly modulated by an oncogenetic gene, miR-362-5p.

Lastly, we will discuss the category “regulation of key signaling pathways and actin cytoskeletal pathways” based on the expression profiles of PCNA, CDK4, cyclin D1, and p21 of THP-1 cells following silencing of miR-362-5p or induction of GAS7. Previous work has revealed that, some events, such as Stat5-, AKT-, Erk-, and CYFIP1-mediated signaling pathways can be affected by GAS7 in some particular types of cells [34, 35]. Chang et al. [34] indicated that p53 upregulate GAS7, thus attenuating breast cancer cells metastasis through regulating CYFIP1 and WAVE2 complex. On the contrary, GAS7 has a pro-proliferative role in pre-B acute lymphoblastic leukemia cells, possibly through regulating Stat5, AKT, and Erk proliferation signals [35, 36]. GAS7 is also well known for its regulatory effect on actin cytoskeleton reorganization [37]. Interestingly, Reshetnikova et al. [38] pointed out that actin cytoskeleton-dependent G0/G1 cell cycle arrest may be associated with the CDK/cyclin pathway. As indicated from the above discussion, these examples may help us to determine whether the cell proliferation and cell cycle regulators are directly regulated by GAS7 axis in future study.

In summary, we have shown that GAS7, a direct downstream target of miR-362-5p, can exert its tumor suppressive functions in THP-1 cells through regulation of PCNA, CDK4, cyclin D1, and p21. Our results suggest that suppression of miR-362-5p may have therapeutic potential in treatment of AML.

## Electronic supplementary material

Below is the link to the electronic supplementary material.

Supplementary material 1 (DOCX 19 kb)

## Abbreviations

AML: Acute myeloid leukemia
GAS7: Growth arrest-specific 7
CCK-8: CCK-8
ATCC: American type culture collection
FBS: Fetal bovine serum
PVDF: Polyvinylidene difluoride
ECL: Enhanced chemiluminescent
IHC: Immunohistochemistry
