# Supplementary material for: miR-362-5p promotes cell proliferation and cell cycle progression by targeting GAS7 in acute myeloid leukemia
Source: Hum Cell. 2020 Jan 10;33(2):405–15. doi: 10.1007/s13577-019-00319-4 (PMC7080691; doi:10.1007/s13577-019-00319-4)
Supplement: Supplementary file 1 — Supplementary material 1 (DOCX 19 kb) [file 13577_2019_319_MOESM1_ESM.docx]

**Supplementary Table 1: Basic clinicopathological characteristics in 24 patients with acute myeloid leukemia**

| **Number** | **Age** | **Gender** | **Initial WBC (×10^9^/L)** | **HGB (g/L)** | **PLT (×10^9^/L)** | **FAB subtype** | **Relapse** | **CR** |
| --- | --- | --- | --- | --- | --- | --- | --- | --- |
| 0818632 | 44 | Female | 42 | 56 | 14 | M1 | No | Yes |
| 0816123 | 45 | Male | 30 | 47 | 63 | M4 | No | No |
| 0716731 | 46 | Male | 100 | 88 | 26 | M5 | No | No |
| 1016157 | 28 | Female | 120 | 75 | 55 | M7 | Yes | No |
| 0742012 | 52 | Female | 86 | 96 | 33 | M3 | Yes | Yes |
| 1107026 | 63 | Male | 21 | 62 | 54 | M1 | No | Yes |
| 1132654 | 53 | Female | 46 | 46 | 46 | M3 | No | No |
| 0845321 | 54 | Male | 75 | 45 | 63 | M5 | No | No |
| 1163245 | 56 | Male | 53 | 94 | 35 | M1 | Yes | Yes |
| 0607903 | 56 | Female | 46 | 107 | 71 | M5 | Yes | No |
| 0815095 | 57 | Female | 70 | 44 | 83 | M1 | No | No |
| 1002438 | 57 | Male | 150 | 123 | 69 | M6 | No | Yes |
| 1563285 | 72 | Male | 112 | 35 | 47 | M1 | Yes | No |
| 1745625 | 39 | Female | 52 | 112 | 70 | M2 | No | No |
| 4321678 | 68 | Male | 47 | 83 | 12 | M3 | Yes | No |
| 3652194 | 59 | Male | 102 | 68 | 63 | M1 | Yes | No |
| 1346853 | 70 | Male | 142 | 89 | 46 | M3 | No | Yes |
| 2354163 | 38 | Female | 135 | 63 | 43 | M2 | No | No |
| 0865432 | 56 | Male | 142 | 96 | 37 | M5 | No | No |
| 2563419 | 47 | Male | 12 | 59 | 35 | M1 | Yes | Yes |
| 4635218 | 65 | Male | 43 | 89 | 68 | M5 | No | No |
| 4653182 | 69 | Female | 45 | 57 | 23 | M2 | Yes | No |
| 1356241 | 64 | Male | 50 | 95 | 76 | M3 | No | Yes |
| 2034126 | 63 | Female | 71 | 65 | 18 | M2 | Yes | No |

WBC white blood cells; HGB (g/L); PLT platelet; FAB French–American–British; CR complete remission
